# Primer‐Disk‐Enabled DNA Data Storage System with Index and Record‐Many‐Read‐Many Features

**DOI:** 10.1002/advs.202502367

**Published:** 2025-06-04

**Authors:** Jiaxiang Ma, Yu Yang, Ben Pei, Shengli Mi, Zhuo Xiong, Liliang Ouyang

**Affiliations:** ^1^ Department of Mechanical Engineering Tsinghua University Beijing 100084 China; ^2^ State Key Laboratory of Tribology in Advanced Equipment Tsinghua University Beijing 100084 China; ^3^ Division of Advanced Manufacturing Graduate school at Shenzhen Tsinghua University Shenzhen 518055 China

**Keywords:** DNA data storage, inkjet printing, micro‐fabrication, record‐many‐read‐many memory

## Abstract

DNA data storage has emerged as a promising information storage technology by encoding information down to base molecules. However, it remains a challenge to structure the DNA data with ease of recording, retrieving, and reading. Here, a primer‐disk‐enabled hierarchical DNA data storage system is introduced, which allows for the multiple immobilizations of DNA molecules and the generation of corresponding QR codes for retrieving. The primer disk is pre‐engineered to present multiple primers, on which encoded DNA molecules with complementary primers can be covalently immobilized on demand via solid‐phase PCR. Each DNA file can be retrieved by inkjet printing a fluorescent QR code. A primer disk with up to 10 primers is used. The results show that different DNA files can be subsequently stored on the disk. One can have readily access to the index via fluorescent QR codes and decode information after sequent imaging, convention, and recognition. To this end, the recorded DNA files can be randomly read via solid‐phase PCR with sufficient copies of collected DNA for up to 20 reads. Together, this work provides a new DNA data storage system with index and record‐many‐read‐many features, paving the way for the practical use of DNA data storage.

## Graphical abstract

A primer‐disk‐enabled DNA data storage system is introduced, which allows for append‐recording of DNA files on demand, retrieval via inkjet printed QR code, and multiple ramdom read of DNA information via solid‐phase PCR. The hiearchical system with index and record‐many‐read‐many features paves the way for the practical use of DNA data storage.

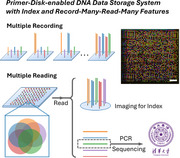

## 1 Introduction

The annual size of global data keeps growing and is expected to reach 175 ZB by 2025.^[^ ^1^ ^]^ However, traditional storage media such as compact disc read‐only memory (CD‐ROM) and hard disks are reaching their physical limit regarding information density. Using molecules to store information could break this limit, with deoxyribonucleic acid (DNA) as one of the most promising candidates. Nature has stored genetic information in DNA, which has been encoded with sophisticated information in the base sequence. The development of de novo DNA synthesis technology makes it possible to create artificial DNA molecules for customized data storage. DNA data storage has attracted much attention recently due to its advantages over traditional silicon‐based systems, including high storage capacity, long‐term retention, and low maintenance cost.

The field of DNA data storage has made significant progress in various fundamental aspects, including encoding/decoding,^[^ ^2^, ^3^, ^4^ ^]^ synthesis,^[^ ^5^, ^6^ ^]^ and sequencing.^[^ ^7^, ^8^, ^9^ ^]^ For example, Church et al. introduced additional restrictions in their coding scheme to eliminate homopolymers.^[^ ^10^ ^]^ Later, the DNA Fountain algorithm was introduced with eliminated homopolymers while maintaining a considerable information density (1.57 bits nt^−1^).^[^ ^3^ ^]^ To further minimize the decoding failure, Yin‐yang code was developed to provide dynamic combinatory coding schemes that can eliminate homopolymers, maintain information density, and recover data stably.^[^ ^2^ ^]^ The development of large‐scale de novo DNA synthesis technology ^[^ ^11^, ^12^ ^]^ has enabled researchers to develop various synthesis approaches for DNA data storage. Antkowiak et al. demonstrated a DNA storage system based on the massively parallel light‐directed synthesis of DNA, which was considerably cheaper than conventional solid‐phase synthesis.^[^ ^5^ ^]^ Nguyen et al. developed a nanoscale DNA storage writer, which further scaled DNA write density to 2.5 × 10^7^ sequences per square centimeter.^[^ ^13^ ^]^ In another example, Lee et al. reported a multiplexed enzymatic DNA synthesis method for DNA data storage.^[^ ^6^ ^]^ Enzymatic synthesis of DNA has emerged as a promising alternative to chemical synthesis, and the aqueous environment essentially matches the workflow of DNA data storage.^[^ ^6^, ^14^ ^]^ For sequencing, most researchers use high‐throughput sequencing technologies. Xu et al. integrated synthesis and sequencing on a single chip to achieve an efficient and practical DNA data storage system.^[^ ^8^ ^]^

Despite the progress, more efforts are needed in DNA data storage management toward practical application. Random access is a significantly desirable feature in DNA data storage systems, especially when the data pool is large. To this end, the incorporation of the index and encode‐derived configuration information into the storage system and their easy access are essential. Previous work can retrieve special files through DNA position separation,^[^ ^15^ ^]^ polymerase chain reaction (PCR)‐based methods,^[^ ^16^ ^]^ or physical separation of the PCR reaction.^[^ ^17^, ^18^ ^]^ In a recent example, Choi et al. developed a DNA micro‐disk made of DNA‐embedded polymers and used optofluidic lithography technology to pattern a QR code on the disk for DNA data retrieval. This innovative method can record a large number of index information beyond simple tags on each file, without the need for other systems to store index information. The write‐once‐read‐many (WORM) memory feature^[^ ^19^ ^]^ was successfully demonstrated with up to 20 reads, each of which was performed by PCR. However, the immobilized DNA inside the disk can't be changed easily and the index is fixed. For such non‐append‐recording mode, a new data record involves the preparation of a new disk, which would cause a waste of storage space and time. For example, long‐term archives always require version control for data provenance because files could have different versions, where recordable media would be beneficial. Recording DNA file one by one with temporal separation on demand could innovate the way one manages DNA storage, similar to recordable silicon‐based media (e.g., recordable tape and digital versatile disc), allowing append operations to data already on the carrier. Newman et al. arranged multiple spots on a single glass plate to store large amounts of data,^[^ ^15^ ^]^ while Sadremomtaz et al. highlighted the scalability of on‐demand information storage.^[^ ^20^ ^]^ Rewriting is another way to achieve the append‐recording of DNA information, either using DNA‐related enzymes or taking advantage of the complementary pairing and strand replacement properties of DNA.^[^ ^9^, ^21^, ^22^, ^23^, ^24^, ^25^, ^26^, ^27^ ^]^ Despite the progress, the combination of appending recording and multiple readings while maintaining retrieving is yet to be achieved in a DNA data storage system.

**Figure 1:**
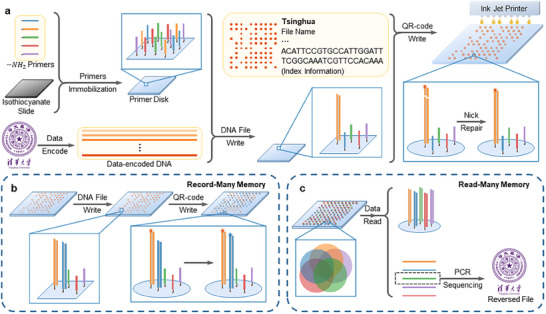
The design of the primer disk enables record‐many and read‐many features. a) The schematic of primer disk preparation and information recording process. Various primers were covalently immobilized to an isothiocyanate slide to form a primer disk. Targeted file information was encoded into many DNA sequences using yin‐yang code, with 20‐nt forward and reverse primers flank these sequences. Next, data‐encoded DNA is synthesized using de novo synthesis techniques. The DNA file is formed by extending the complementary primer on the primer disk by solid‐phase PCR. The QR‐code dot array is formed by attaching fluorophores to the ends of DNA files using T4 DNA ligase. b) The schematic of the multiple‐recording process. Different DNA files were formed by extending the corresponding DNA molecules from different primers on the primer disk. The QR codes were subsequently written by inkjet printing different fluorescent molecules. c) The schematic of multiple reading processes. Files were recovered by producing DNA copies from the immobilized DNA molecules via solid‐phase PCR on the primer disk, followed by DNA sequencing.

In this study, we establish a primer‐disk‐enabled DNA data storage system with index and record‐many‐read‐many (RMRM) features. The innovative primer disk serves as a storage carrier in the presence of multiple primers covalently bonded on the surface. DNA data writing is achieved by binding desired DNA molecules with a specific complementary primer to the primer disk, forming a DNA file. The binding of DNA molecules to the disk does not involve additional modification to the DNA and only needs regular PCR. The corresponding index is encoded and stored into a quick response (QR)‐code dot array on the surface by on‐demand inkjet printing fluorescence molecules and T4 DNA ligase (**Figure** **1**a). The fluorescence molecules can easily bond to the end of the corresponding DNA molecules via T4 DNA ligase on site within minutes. By repeating this process with different DNA molecules and primers, multiple recordings of different DNA files and data‐specific indexes into a single disk can be achieved (Figure 1b). Guided by the QR‐code index, DNA data reading is achieved by reproducing sufficient copies of DNA of interest via solid‐phase PCR in situ and subsequent sequencing (Figure 1c). When a file on a primer disk needs to be read, one obtains the index information of the file by fluorescently scanning the QR‐code dot array and then amplifies the DNA in situ with the corresponding primer. After sequencing the DNA and decoding the sequence, the data could be obtained. This reading approach allows for random and multiple access to the specific data on demand without losing information. Taken together, using our RMRM strategy, DNA data can be appended additively, read multiple times, and accessed randomly.

## 2 Results and Discussion

### 2.1 Multiple Specific Immobilization of DNA on Primer Disk

**Figure 2:**
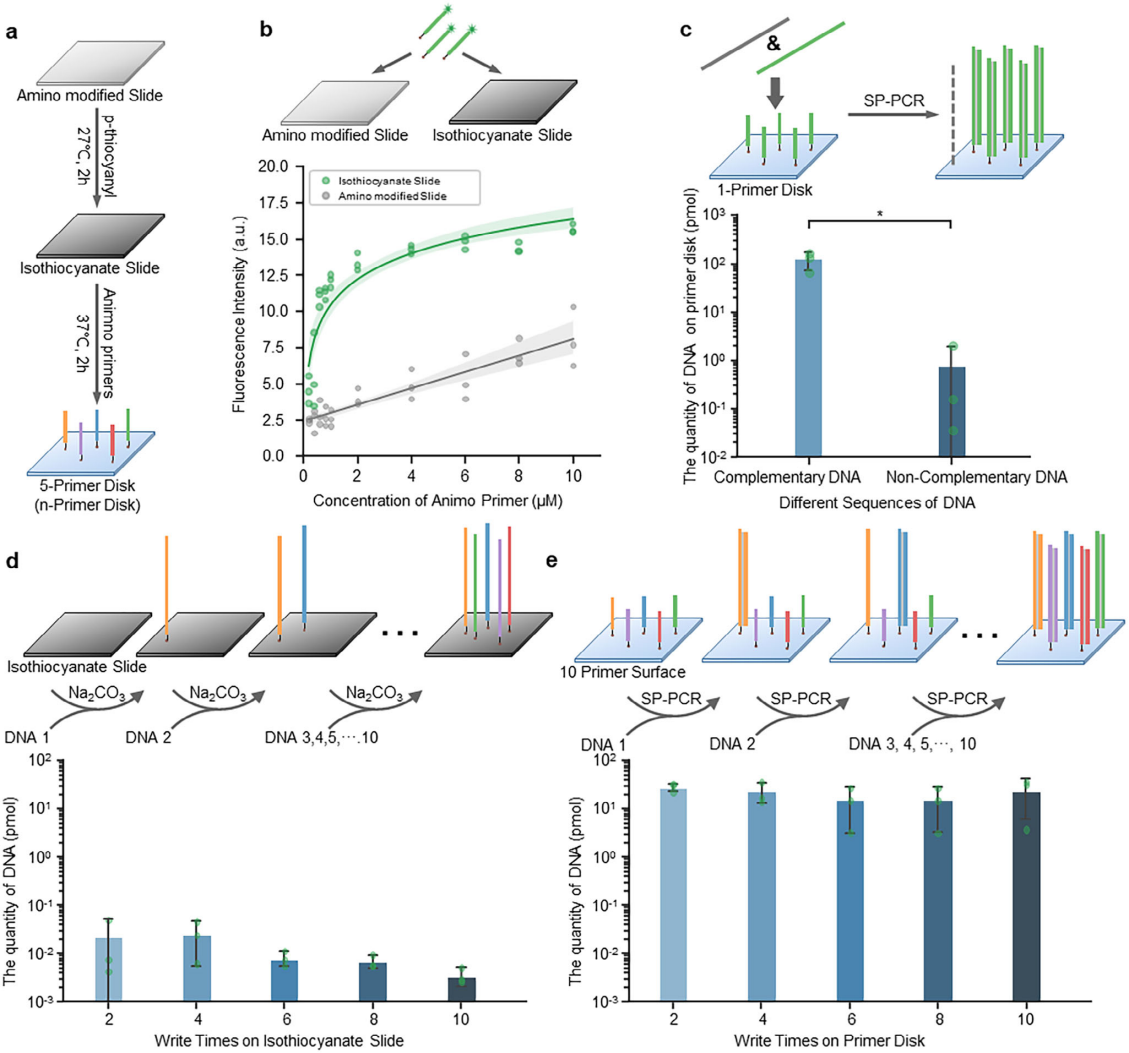
Preparation of primer disk and specific DNA immobilization on primer disk. a) Preparation process of a primer disk includes the amino modification, isothiocyanate modification, and subsequent immobilization of different 35‐nt 5´‐amino‐modified ssDNA. The schematic indicates five distinct primers immobilized on the surface with different colors. b) The immobilization of ssDNA amino primers on amino‐ and isothiocyanate‐modified slides under different concentrations (N = 3). The 3′ end of the ssDNA was connected to the FAM group for fluorescence detection. The continuous lines are fitting curves of the experimental data points. c) The quantity of DNA immobilized on a 1‐primer disk with specific or non‐specific DNA templates by qPCR (Mean ± SD, N = 3). d) The immobilization of data‐encoded amino‐DNA directly on isothiocyanate slides. The process was repeated 10 times at different positions of the same isothiocyanate slide. Quantification of DNA immobilized on the isothiocyanate slide was determined by qPCR (Mean ± SD, N = 3). e) Ten different sequences of DNA were immobilized on a 10‐primer disk subsequently. The copies were collected by solid phase PCR and the quantity of DNA immobilization was determined via qPCR at each cycle (Mean ± SD, N = 3).

The primer disk is created by conjugating specific primers to a glass slide. To do so, an amino‐modified slide was treated with p‐phenylene di‐isothiocyanate to covalently immobilize isothiocyanate groups on the surface. Specific 5′‐NH_2_‐modified solid‐phase primers (primer sequences can be found in Table S1, Supporting Information) were subsequently immobilized to the isothiocyanate‐modified slide, forming the so‐called *primer disk*. X‐primer disk indicates a primer disk containing X types of distinct primers on the surface (**Figure** **2**a; Figure S1a, Supporting Information). After modification, the contact angle of the slide increased significantly, suggesting the successful connection of the isothiocyanate groups (Figure S1b, Supporting Information). To verify the binding efficiency of primers on the slides, we incubated isothiocyanate‐modified and regular amino‐modified slides with primers of different concentrations. The primers were conjugated with the FAM group to determine the relative binding efficiency via fluorescence detection (Figure S2, Supporting Information). The results indicate that the number of primers bonded on the isothiocyanate slide presented a burst increase with the increase of primers up to 2 µm, and then approached a steady level with the continuous increase of primers, suggesting a saturation state of isothiocyanate‐amino reactions (Figure 2b). This result was consistent with the literature, which reported an optimized concentration of amino‐modified primers to be ∼2 µm when bonding to isothiocyanate surface.^[^ ^28^ ^]^ Thus, we used 2 µm primers to prepare the primer disk. In contrast, the amount of primers bonded on the amino slide increased linearly with the input concentrations, indicating less efficient binding via electrostatic adsorption. Overall, the optimized isothiocyanate slide supported the binding of much more (∼3 folds) primers than the regular amino slide did (Figure 2b). Then we created a 1‐primer disk based on the isothiocyanate slide and tested the specific immobilization of DNA data. The DNA molecules with a complementary primer sequence achieved a much higher binding efficiency than those with random sequences did. Through qPCR, we found the collected DNA amount via solid‐phase PCR with the complementary primer group was two orders of magnitude higher than the non‐specific counterpart (Figure 2c). These results confirm the high efficiency of primer immobilization and specific recording of DNA files based on the given primer sequences.

Before verifying the multiple recording of DNA files on the established primer disk, we first test whether multiple DNA immobilizations could be realized directly on an isothiocyanate slide. We subsequently introduced the same DNA file solutions to ten different positions on the same isothiocyanate slide. Between adjacent recording steps, the slide was washed with ammonium hydroxide throughout the surface. The DNA quantification assay shows that the amount of immobilized DNA from each file was generally low and dropped with time (Figure 2d), which indicates that the multiple immobilizations of DNA directly on the isothiocyanate slide are impossible. This is probably because of the instability of isothiocyanate, which may be oxidized in the air with time. Here, we presented a storage media named primer disk, where primers (oligonucleotides) are presented on the surface by reacting with the isothiocyanate groups. With the complementary base pairing principle, DNA files can be stored by specific primers on the disk.^[^ ^28^ ^]^ To demonstrate this, we made a 10‐primer disk and immobilized 140‐nt DNA files corresponding to different primers one by one for 10 cycles. We collected the copies of DNA bonding to the primer disk via solid‐phase PCR and qPCR. The results indicate that the quantities of different 140‐nt DNA molecules from each recording maintain a considerable level and do not change much during 10 cycles (Figure 2e). This also confirms that the potential reduction of hybridization efficiency induced by left‐over DNA strands does not affect the detection for at least 10 cycles. Moreover, the storage capacity could possibly be further enhanced by incorporating methods like nanocones^[^ ^29^ ^]^ and 9G DNA chips,^[^ ^30^ ^]^ which can generate ample space on the surface for each recording. In conclusion, our primer‐disk approach allows for multiple recordings of DNA files, and the number of recordings mainly depends on the number of primer types on the disk.

### 2.2 Writing of Fluorescent Dot Array as Index

To realize the writing of the index into a QR code and integrate this function into the primer disk system, we used a high‐resolution inkjet printing system to additively create a fluorescent dot array. Fluorescence has a long lifetime and different fluorescent dyes could be easily distinguished using a multichannel fluorescence detector, increasing the storage density.^[^ ^31^ ^]^ The ease of quenching and removal of fluorescent signals also add to the opportunity for re‐writing. To attach the fluorescent dyes to DNA molecules on the prime disk, we modified a short DNA with fluorescent dyes and used T4 DNA ligase to repair DNA nicks and ends.

**Figure 3:**
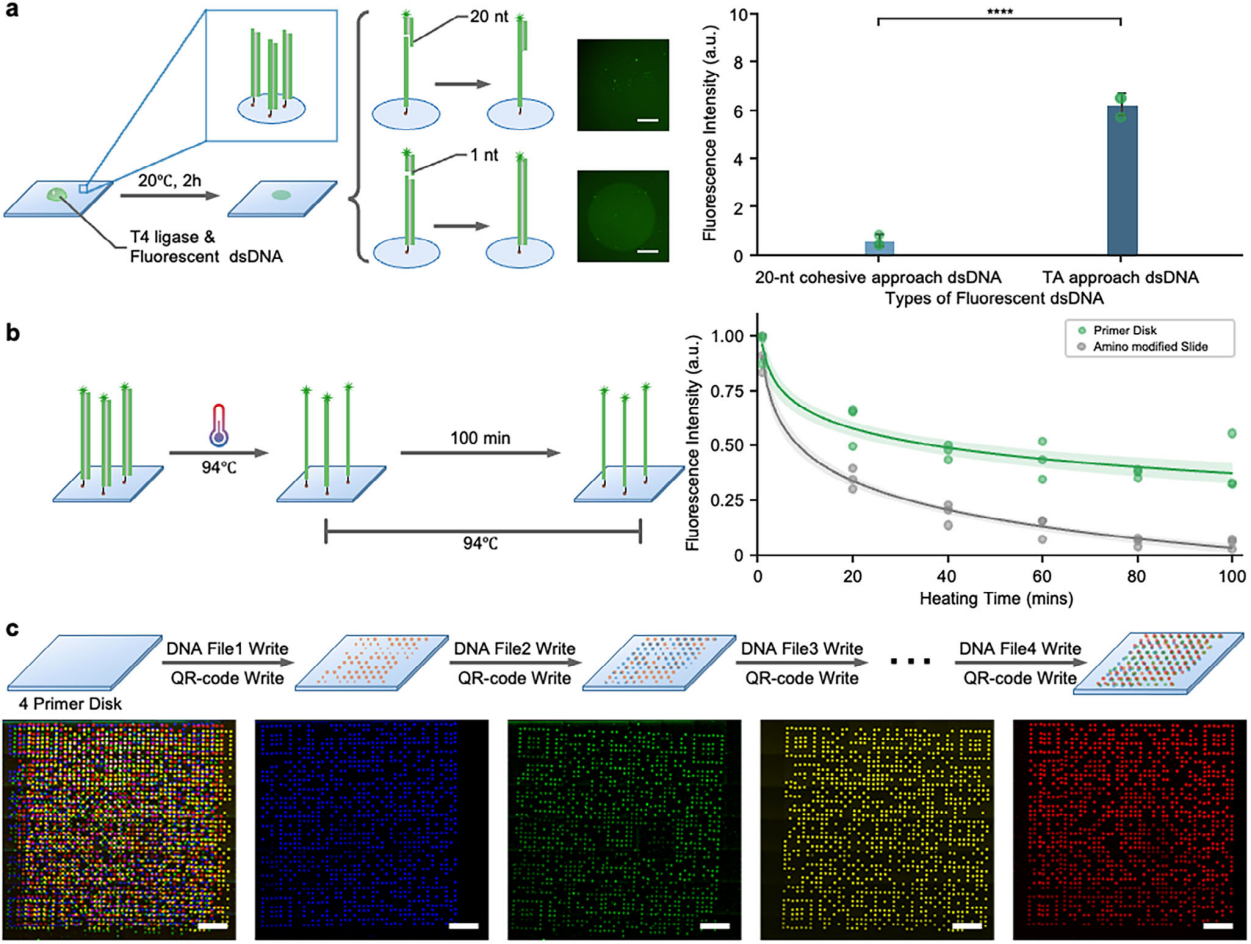
Inkjet Writing of fluorescent QR‐code dot array. a) Connection efficiency of two types of fluorescent dsDNA on a disk recorded with data‐encoded DNA. The 20‐nt cohesive approach uses the nick repair properties of T4 DNA ligase, while the TA approach uses the property that T4 DNA ligase could connect single base overhangs of dsDNA, both involve a nick repair process afterward (Mean ± SD, N = 3). b) The heat stability of fluorescent dyes immobilized on the data‐recoded disk via TA approach over 100 min. In the control group, fluorescent dsDNA was immobilized to amino‐modified slides via physical absorption (N = 3). c) Four DNA files were recorded to the 4‐primer disk together with corresponding index dot array printing. Scale bars: 500 µm.

We first compared the efficiency of binding two types of fluorescence‐modified DNA on data recorded disk using T4 DNA ligase (Figure S3, Supporting Information). One approach is to bind fluorescence‐modified dsDNA to the ssDNA file via 20‐nt base pairing (**Figure** **3**a). Previous work has shown that ligation of a dsDNA with an overhang to a complementary ssDNA fragment requires a minimum five base pair duplex. In this approach, there were 20 base pair duplexes.^[^ ^32^ ^]^ However, it was found that ssDNA on the primer disk could not effectively connect fluorescent dsDNA through T4 DNA ligase, which was likely due to the inability of the ssDNA secondary structure to expose the reaction site. Here, we designed a surface reaction connection approach by taking advantage of the property of Taq DNA polymerase that adds base A to the end of DNA. The fluorescence‐modified dsDNA binds to the dsDNA file via 3′‐A overhangs generated by Taq DNA polymerase during PCR (Figure 3a). The results indicate that our TA binding approach can effectively attach fluorescent dyes to DNA molecules. The 20‐nt cohesive approach showed a fluorescence intensity of ∼0.56 (a.u.), while the TA binding approach showed a fluorescence intensity of ∼6.22 (a.u.), with a significant difference (Figure 3a). Meanwhile, once a DNA file was written, 3′‐A overhangs were generated by Taq DNA polymerase. Then, the fluorescent dsDNA could bind to the dsDNA file, forming different fluorescent dot Arrays.

After binding the fluorescence on the data disk, we further verified the thermo‐stability of fluorescent dyes in the liquid buffer since the subsequent PCR process involved high temperature (i.e., 94 °C). The results demonstrated that the fluorescent signals retained 40–50% of the initial intensity throughout the 100‐min incubation at 94 °C after the first wash, which might remove some excess dyes that were not covalently immobilized (Figure 3b). In the control group, fluorescent dsDNA was immobilized to amino‐modified slides via physical absorption. The results indicated a continuous decrease of fluorescence intensity over the 100‐min incubation, and less than 5% intensity remained after 80 min for the control group (Figure 3b). Thus, our approach with TA pairing and T4 ligase nick repair could stably immobilize fluorescent dyes on DNA data disk.

Next, we explored the subsequent writing of fluorescent dot arrays through inkjet printing on a 4‐primer disk. In practical use, the sequences of the four primers are pre‐determined and marked on the disk, indicating the specification and storage capacity (e.g., 4X records). Data‐encoded DNA is synthesized with a complementary primer end (Figure S4, Supporting Information). Each DNA file writing is followed by the corresponding QR‐code writing with a dot distance of 84 µm with an on‐demand inkjet printer (Figure S5, Supporting Information), forming a hierarchical data storage set. The disk was placed in a suitable humidity environment (50% humidity) to ensure that the reaction was carried out successfully (Figure S6, Supporting Information). Four different data sets (Files No. 1–4 as shown in Table S2, Supporting Information) were subsequently recorded on this 4‐primer disk. The fluorsecent images clearly showed that four arrays could be divided by different fluorescent channels, and they did not interfere with each other (Figure 3c), in consistent with literature.^[^ ^31^ ^]^ Using the spectral phasor analysis technology,^[^ ^33^ ^]^ the storage density of index information and the number of dot arrays can possibly be further increased.

### 2.3 Multiple Recording, Reading, and Random Access to DNA Files

**Figure 4:**
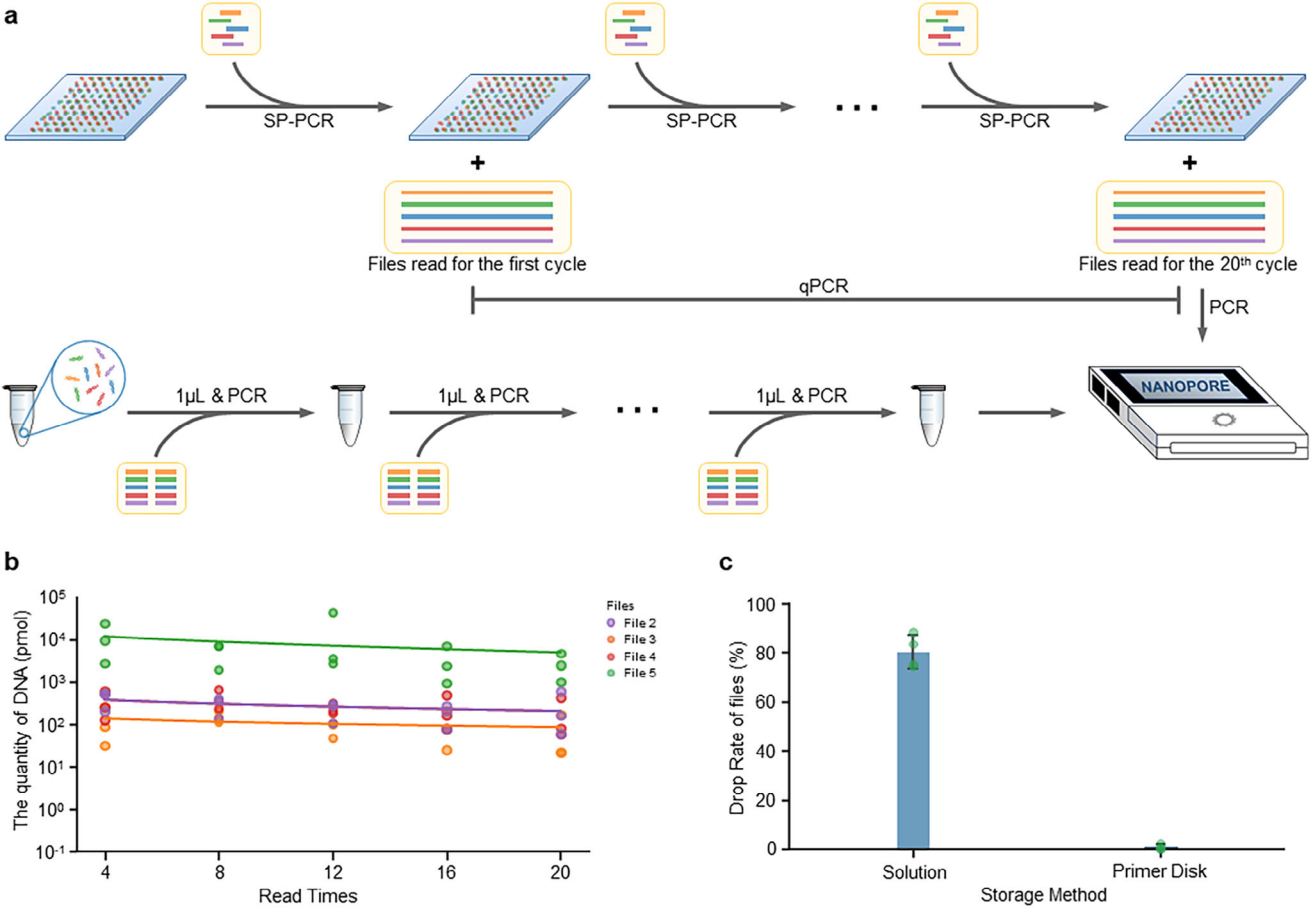
Multiple random readings of the DNA files on the primer disk. a) Schematic of multiple reading processes via solid‐phase PCR with reverse primers and pfu DNA polymerase and nanopore sequencing. The control is set to store DNA in water and experience similar multiple reading processes. b) The production of the DNA files over 20 readings for different files. c) The loss rate of individual files after 12 rounds of readings using our primer‐disk approach and traditional bulk approach in solution (Mean ± SD, N = 4).

To read the data stored in DNA files on the disk, we designed a standard reading scheme. We first checked the fluorescent QR code and collected index information. Solid‐phase PCR was performed to collect desired DNA copies. To read specific data from the DNA data pool, conventional schemes usually separate DNA datasets and perform PCR in liquid. Our approach does not need to separate the datasets physically and is free of extra PCR to recover the DNA. To demonstrate this in our storage system, four files (Files No. 2–5 as shown in Table S2, Supporting Information) with a total size of 144 KB were encoded and stored in 7323 DNA sequences (length of ≈140 bases) using the previously reported method.^[^ ^2^ ^]^ The primer sequences of these files can be found in Table S2 (Supporting Information), while the original files and their encoded DNA sequences are provided as supporting information. Each file consists of ≈2000 distinct sequences fixed on the primer disk as described in this manuscript. Then PCR was performed to amplify the DNA files on the primer disk, followed by nanopore sequencing (**Figure** **4**a). The measurement of DNA production indicates that a considerable amount of DNA can be collected throughout the 20 readings, which lays the basis for highly reproducible data reading. This is confirmed by selectively collecting and reading specific files (Figure 4b). To make a fair comparison, we simultaneously amplified the same DNA files in the solution as a control group. The file loss rate was determined by DNA sequencing. After 12 rounds of reading, the average loss rate of the four files was only ∼1% for the primer disk approach, while the loss rate of DNA amplification in solution was as high as ∼80% (Figure 4c). These results clearly showed that the DNA files on the primer disk were more stable with much lower data loss rates than in solution. The primer‐based DNA file fixation and subsequent solid‐phase PCR reading optimize the problem of PCR bias, with a significant difference in the distribution of read length compared to the traditional solution amplification approach (Figure S7, Supporting Information).

### 2.4 Rewriting of Index Dot Array

Although multi‐channels of fluorescence can be resolved using spectral techniques, the differentiation resolution is limited, thus leading to finite times of appending writing. Here, we also tried to use restriction enzymes to excise the fluorophores without affecting the DNA files. To achieve this, we designed the TA end‐linked DNA by taking into account the avoidance of degenerate sequences during DNA coding. We excised the fluorophores using the restriction enzyme Sma I, followed by multiple writings of the DNA information and the QR code information. At the same time, fluorescent dsDNA can also be excised using restriction enzymes. Unlike other fluorescence elimination methods, this method won't cause damage to the file database while ensuring the stability of index information storage.

**Figure 5:**
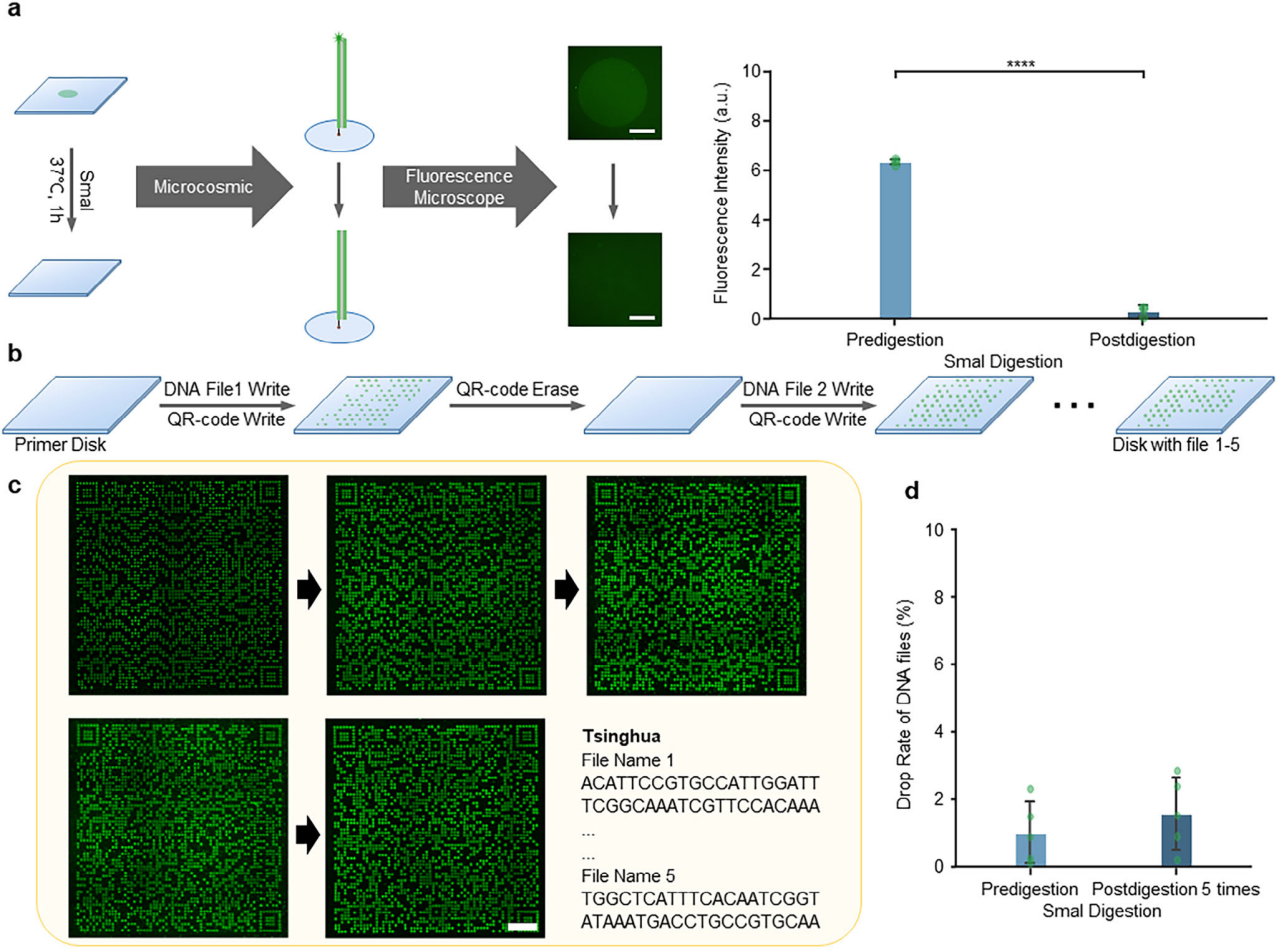
Rewriting Index Dot Array via SmaI erasing. a) The fluorescent group immobilized on the data‐recorded disk was removed via treatment with enzyme Sma I (Mean ± SD, N = 3). b) Repeat writing of QR codes for 5 DNA files, following writing and erasing processes for each file. c) Five fluorescent dot matrices are subsequently written on the same primer slide, with Sma I erasing in between. d) The DNA file loss rates before and after excision (N = 5). Scale bars: 500 µm.

We performed the excision experiment as shown in **Figure** **5**a. The fluorescent dyes were fixed on the disk via fluorescent dsDNA, and the dsDNA can be successfully excised through Sma I. Then, we performed a rewritable demonstration based on a 5‐primer disk. Before writing a new DNA file set (i.e., data‐encoded DNA recording plus index dot array writing), excision of the previous fluorescent dot matrix was performed (Figure 5b; Figure S8, Supporting Information). Then, a new DNA file was recorded and the new index dot array was written (Figure 5c). Each fluorescent index dot array was presented clearly with interruption over the five cycles of writing‐erasing. The details of the five files are attached in Figure S9 and Table S2 (Supporting Information). We analyzed the error rate of conversion of fluorescent dot code (Figure S10, Supporting Information) and we analyzed the file loss rates of DNA files through DNA sequencing to assess the side effects of restriction enzymes on DNA files. The results demonstrated negligible loss of DNA data files with the use of enzyme Sma I (Figure 5d). Combined with the DNA coding process, the excision only occurs at fluorescent dyes and does not affect DNA files.

## 3 Conclusion

In this paper, a primer‐disk‐enabled DNA data storage system is designed and established to address the implementation challenges of DNA data storage, especially the multiple recordings and multiple readings with index. We combined a surface droplet reaction based on T4 DNA ligase and inkjet printing technology to stably immobilize data‐encoded DNA and pattern index dot array, respectively. Such a hierarchical data storage system allows us to rapidly check the index via imaging and selectively read DNA files by sequencing. We have demonstrated the storage capacity of 10 files in the current study. It is worth noting that the design of primers is ultimately the most critical factor in determining the number of files to be stored and distinguished. Our results demonstrate that the primer disk has high specifications and a consistently high binding rate over multi‐recording process regarding the immobilization of data‐encoded DNA. Index QR code arrays can be covalently immobilized on the data‐recorded disk with high resolution (∼40 µm of dot diameter and 84 µm of dot distance) in a merge mode without interruptions. We have also demonstrated random access to the written data up to 20 times with negligible decrease in data collection quantity and file loss. The process of erasing the QR code dot array is also realized by restriction endonuclease, and re‐writing was realized by Taq enzyme. Together, our work establishes a DNA information storage and management system that integrates essential data storage features, including multiple recording, multiple reading, and random access.

Compared with the previous work, our work requires easier steps and less materials. Meanwhile, we increase the flexibility of the system, so it can realize the record‐many feature. Ideally, the storage density of our system can reach 10^12^ bit/mm^2^, comparable to existing work (Table S3, Supporting Infromation). It is worth noting that our study contributes little to DNA synthesis technology, one of the most significant bottlenecks for DNA data storage, but rather focuses on providing a DNA data storage management system with Record‐Many‐Read‐Many features. With the development of low‐cost DNA synthesis technology in the future, our approach could be readily used affordably. Compared to existing DNA micro‐disk work with Record‐Once‐Read‐Many,^[^ ^19^ ^]^ our approach allows for the addition of hierarchical information on demand temporally. The append‐recording mode allows for append operations to data already on the carriers, similar to recordable silicon‐based media. In our current approach, additional imaging of fluorescent QR codes is needed, which can be addressed by using new code systems in the future. Furthermore, our primer‐disk‐enabled DNA data storage system has involved ink‐jet printing and has the potential to be incorporated with the in‐situ synthesis steps in the future. Combined with sequencing technology, this technology might provide an end‐to‐end DNA data storage system.^[^ ^34^ ^]^

## 4 Experimental Section

### Materials

Amine functionalized glass slides (Qiyuebio); Pyridine (99.9%, Macklin); N,N‐Dimethylformamide (99.5%, Aladdin); 1,4‐Phenylendiisothiocyanat (98%, Sigma); Methanol (99.9%, Boer); Acetone (99.5%, TGREAG); Carbonate Buffer (pH = 9.0, Boer); Prehybridizaion Solutions in SSC (pH = 7.0, Boer); Taq DNA Polymerase (Solarbio); Pfu DNA Polymerase (Solarbio); Gene Frame (25 µL, Thermo Fisher); Blunt/TA Ligase Master Mix (NEB).

### Preparation for Primer Disk

Isothiocyanate slides were synthesized according to the method previously reported.^[^ ^28^ ^]^ The amino‐modified slide was immersed in a solution consisting of 0.1 g 1,4‐Phenylendiisothiocyanat, 5 mL pyridine, and 45 mL N,N‐Dimethylformamide, and placed at 27 °C for 2 h in dark. Then the slides were rinsed with N,N‐Dimethylformamide, methanol, and acetone successively for 2 min each. The isothiocyanate slides were finally obtained by blowing dry with nitrogen gas.^[^ ^35^ ^]^ A 25ul Gene Frame was used to cover the isothiocyanate slide. Amino‐modified primers (total concentration of 2 µm and in NaCO_3_ solution with pH = 9.0) were added to the slide, and the reaction was carried out at 37 °C for 2 h. Then the slide was cleaned with 1% ammonia for 5 min, and dried with nitrogen to get the primer disk. The primer disk connection efficiency was characterized using fluorescence microscopy (Oylmpus BX51).

### DNA Oligonucleotide Synthesis

The data‐encoded DNA files were ordered from Genscript, while all DNA oligonucleotides were purchased from Sangon Bio and purified by HPLC. A nuclease‐free TE buffer (10 mm Tris, 0.1 mm EDTA, pH 7.5, Solabio Bio) was used and stored at −20 °C. Details of the synthesized oligonucleotide primers are shown in Table S2 (Supporting Information).

### Solid‐Phase PCR and Product Collection

To record DNA information on the primer disk, data encoded DNA, reverse primers of 0.2 µm and Taq DNA polymerase were added to the primer disk to extend primers on the primer disk. The reaction occurred in a Gene Frame chamber on the primer disk. The amplification scheme was as follows: denaturing at 94 °C for 3 min, secondary denaturing at 94 °C for 30 s, annealing at 55 °C for 30 s, and extending at 72 °C for 30 s. The second denaturation, annealing, and extension processes were repeated for 10 rounds, ending up with an additional 4 min at 72 °C. After cooling down to 4 °C, the slides were immersed in 0.03% SDS 1× SSC solution, and cleaned with 0.2 × SSC (Saline Sodium Citrate buffer) and 0.05×SSC for 5 min to complete the writing of a DNA file.

For the reading on the primer disk, reverse primers of 0.2 µm and Pfu DNA polymerase were added to the primer disk, amplifying DNA on the primer disk. The amplification scheme was as follows: denaturaing at 94 °C for 3 min, secondary denaturing at 94 °C for 30 s, annealing at 55 °C for 30 s, extending at 72 °C for 30 s. Repeat the second denaturation, annealing, and extension process for 2 rounds, and finally extend at 72 °C for 4 min, and collect the product to sequence. The disk was then rinsed three times with 70% ethanol and cleaned once with ultra‐pure water.

### Binding of Fluorescent Dyes to DNA via T4 Ligase

Fluorescent dsDNA with a concentration of 10 µm, T4 blunt, and ddH_2_O were mixed on the ice in a ratio of 2:1:2 and added to the nozzle of the bio‐printer BP4000. The QR code was printed with a resolution of 300 dpi under the humidity of 50%. The printed disk was placed at 20 °C for 1 h, then cleaned with 70% ethanol and ddH_2_O.

### qPCR Detection

qPCR was performed using the CFX96 Touch Real‐Time PCR Detection System (Bio‐Rad). The total reaction volume is 10 µL, consisting of 0.5 µm primer, 1×Bio‐Rad premix, and 1 µL template solution. Initial denaturation is set at 94 °C for 3 min, followed by 40 cycles, 94 °C for 20 s, 55 °C annealing extension for 30 s, and then final denaturation. Fluorescence was measured during each annealing step. To prevent evaporation, the plate was sealed with a clear plate sealant. The CFX Maestro software version 2.2 (Bio‐Rad) was used to perform baseline corrections and calculate threshold cycles (Ct).

### Sequencing with Oxford Nanopore Technologies MinION

Samples were prepared for sequencing according to the protocol of Ligation sequencing Kit (SQK‐LSK110) and sequenced on the MinION(MK1C) sequencer (R9.4.1, all Oxford Nanopore Technologies). DNA ends were repaired via End Repair Buffer (FFPE) and NEBNext Ultra II End Repair/dA Tail Addition Module. Then, samples were purified with AMPure XP magnetic beads. Sequencing adapter F (AMX‐F) and connecting buffer (LNB) were added to the sample, and ligate sequencing adapters supplied to the DNA ends. A long fragment buffer (LFB) or short fragment buffer (SFB) was used to enrich the target sequence length of the test samples. Finally, AMPure XP magnetic beads were used for purification, followed by the addition of sequencing buffer II (SQBII) and loading particle II (LBII) to complete the DNA library construction.

### Excision of Fluorescence via Restriction Enzyme

The excision reaction system was prepared with Sma I, 10× T Buffer, 0.1% BSA, ddH_2_O at a volume ratio of 1:2:2:15. The DNA disk was treated with the excision solution in a 25 µL gene Frame at 22 °C for 1 h. After the reaction, the disk was washed with 70% ethanol three times and ddH2O once.

### Statistical Analysis

The results of all statistics in this paper were obtained from three independent replicates. Python, Scipy, and Seaborn (version 0.13.2) were used for analysis and visualization. T‐test was used to determine the statistically significant difference between different conditions.

## Conflict of Interest

Liliang Ouyang, Jiaxiang Ma, Zhuo Xiong, and Shengli Mi have applied for a Chinese patent relevant to this study (file no. 2024101256907). Other authors declare no conflict of interest.

## Author Contributions

J.M. and L.O. conceived the study. L.O., Z.X., and S.M. supervised the study and obtained funding. J.M. carried out the majority of the experiments and analyzed the data. B.P. designed the DNA immobilization and read‐many scheme. Y.Y. contributed to the DNA sequencing experiments. J.M., Y.Y., and L. O. participated in manuscript writing and editing. All authors reviewed the manuscript.

## Supporting information



Supporting Information

Supporting Information

## Data Availability

The data that support the findings of this study are available from the corresponding author upon reasonable request.
