# Supplementary material for: Primer‐Disk‐Enabled DNA Data Storage System with Index and Record‐Many‐Read‐Many Features
Source: Adv Sci (Weinh). 2025 Jun 4;12(32):e02367. doi: 10.1002/advs.202502367 (PMC12407294; doi:10.1002/advs.202502367)
Supplement: Supplementary file 1 — Supporting Information [file ADVS-12-e02367-s001.docx]

Supporting Information

Primer-Disk-enabled DNA Data Storage System with Index and Record-Many-Read-Many Features

Jiaxiang Ma^a^, Yu Yang^a^, Ben Pei^a^, Shengli Mi^c,^*, Zhuo Xiong^a,^*, Liliang Ouyang^a, b,^*

^a^ Department of Mechanical Engineering, Tsinghua University, Beijing 100084, China

^b^ State Key Laboratory of Tribology in Advanced Equipment, Tsinghua University, Beijing 100084, China

^c^ Division of Advanced Manufacturing, Graduate school at Shenzhen, Tsinghua University, Shenzhen 518055, China

E-mail:

[ouy@tsinghua.edu.cn](mailto:ouy@tsinghua.edu.cn), [xiongzhuo@tsinghua.edu.cn](mailto:xiongzhuo@tsinghua.edu.cn), [mi.shengli@sz.tsinghua.edu.cn](mailto:mi.shengli@sz.tsinghua.edu.cn)

The supporting information contains:

**10 Figures:** Supplementary Figure S1-S10

**3 Tables:** Supplementary Table S1-S3


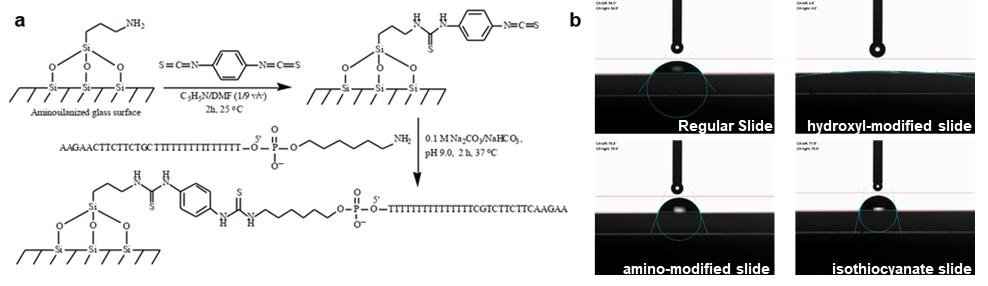


**Supplementary Figure S1. Surface treatment of the slide.**

a. The preparation of the primer disk includes the isothiocyanate modification and binding of ssDNA primers. First, the isothiocyanate group was attached to the amino-modified slide, then amino-modified ssDNA can be attached to the isothiocyanate group. b. Contact angles of the regular slide, hydroxyl-modified slide, amino-modified slide, and isothiocyanate slide.


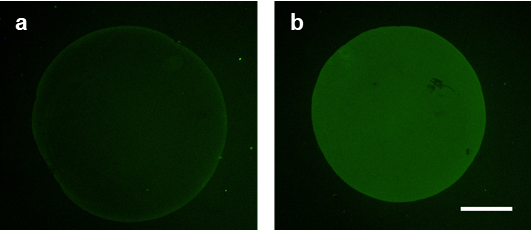


**Supplementary Figure S2. Comparison of electrostatic adsorption and covalent binding.**

The fluorescent images of droplets via dripping fluorescence-labeled primers (2 μM) onto a) regular amino-modified slide and b) isothiocyanate slide. The DNA attached to the amino-modified slide was easily washed away by ammonia, while the DNA attached to the isothiocyanate slide was difficult to remove. Scale bars: 500 μm.


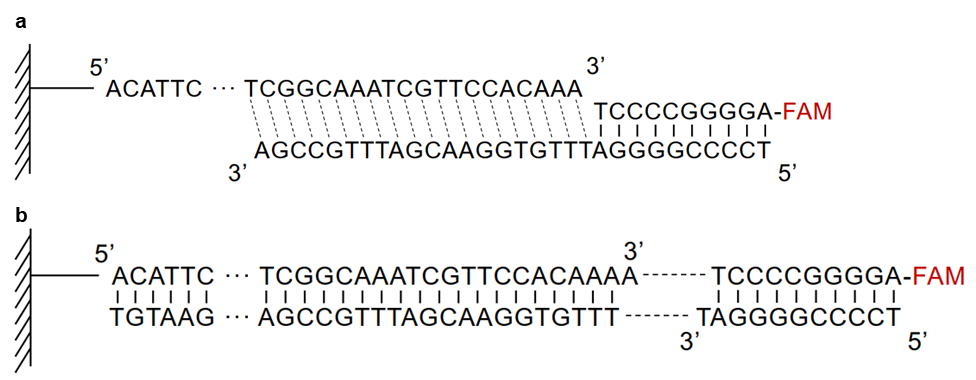


**Supplementary Figure S3. Design of fluorescent dsDNA**.

Two fluorophore bonding schemes we designed. Scheme a) takes advantage of the nick repair properties of T4 DNA ligase. It has previously been verified that T4 DNA ligase can effectively complete nick repair with overhangs of 5-nt or more. The sequences of the overhangs match the reverse primer of the DNA file. Scheme b) takes advantage of the repair properties of T4 DNA ligase on sticky ends and the addition of A to the ends of Taq polymerase during PCR. When the DNA file was written to the primer disk by PCR using Taq polymerase, bases A were added to the ends. The ligation of fluorescent DNA was then completed using T4 DNA ligase.


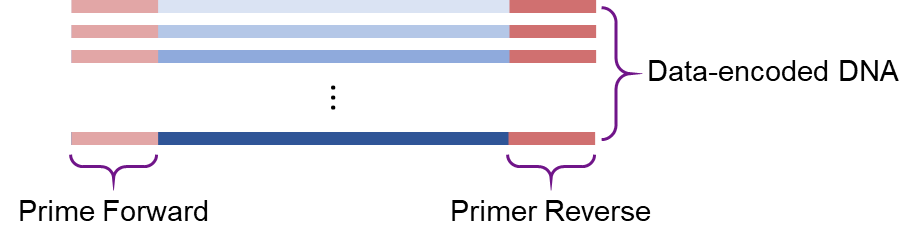


**Supplementary Figure S4**. **Graphical representation of a general DNA file.**

The DNA file consisted of a large number of oligonucleotides with a length of about 140-nt. For each DNA file, the 20 base sequences at both ends were the same, which was called the forward primer and the reverse primer. The base sequences in the middle were different, which were the part that stored information. The forward primer can be paired with the corresponding primer on the primer disk to record the DNA file to the primer disk. Reverse primer and before primer worked together to complete DNA amplification, collection, and sequencing. The middle coding area is generated by the yin-yang code.


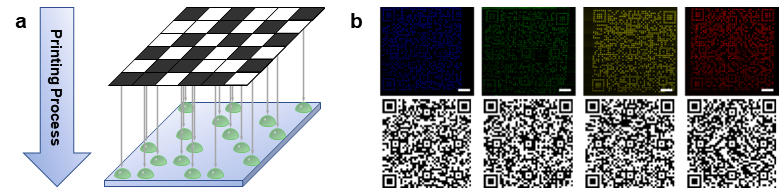


**Supplementary Figure S5. Printing QR-code.**

a. The droplet array was printed according to a binary image, where the black pixel represents a jetting position, and the white pixel represents an empty position. b. Dot arrays of an index for four different files on the same slide and their corresponding QR codes. The QR codes are created by python-qrcode.

**
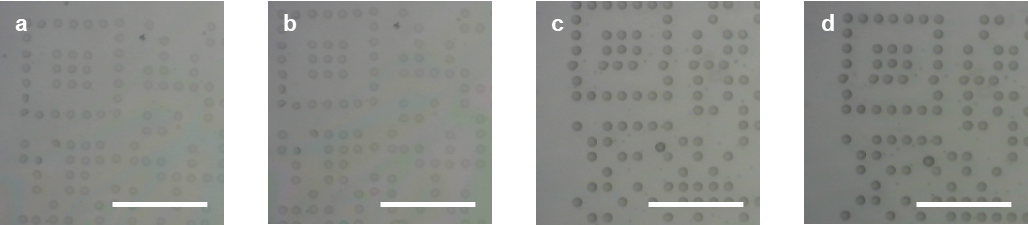
**

**Supplementary Figure S6. Dot array printing of fluorescent dsDNA under different environments.**

Deposit the primer disk at 20 ℃ and 50 % humidity for a) 1h, b) 2h. Deposit the primer disk in a wet box (90% humidity) at 20 ℃ for c) 1h, d) 2h. Scale bar: 500 μm.


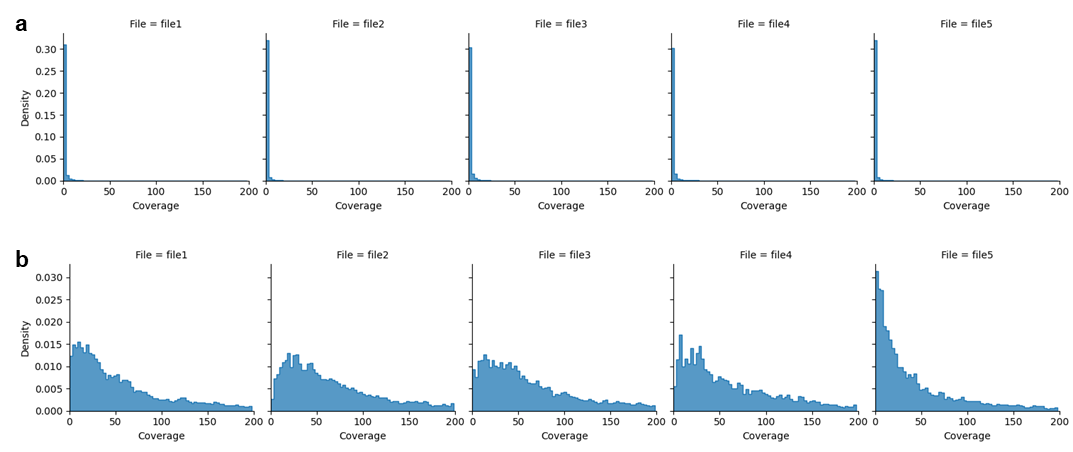


**Supplementary Figure S7. Distribution of read length.**

Read length distribution of DNA sequencing results a) in solution and b) on primer disk at the 10^th^ cycle.


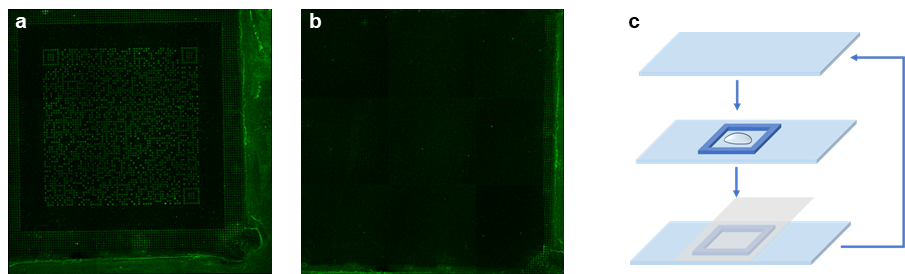


**Supplementary Figure S8. Erasure of dot array.**

a. A representative fluorescent dot array printed on a primer disk (4^th^ recording). b. The fluorescent image of the same array after erasing, indicating the successful erasing. c. a frame was used to form a reaction room on the disk.


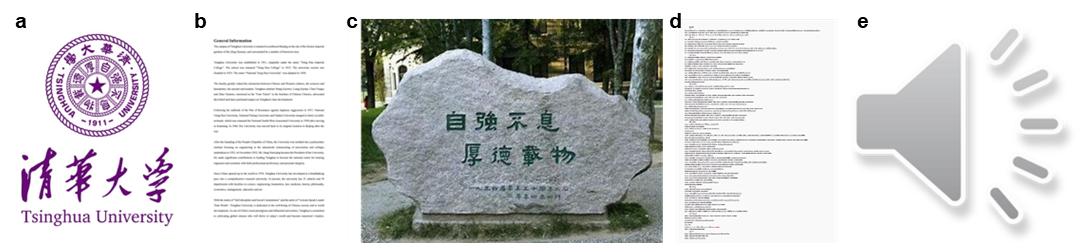


**Supplementary Figure S9.** **DNA files used in this study.**

We encoded 5 files into DNA molecules, which were a) a badge picture, b) a PDF file about Tsinghua general information, c) a stone picture in Tsinghua, d) a Txt file about Tsinghua States, and e) parts of a song, respectively.


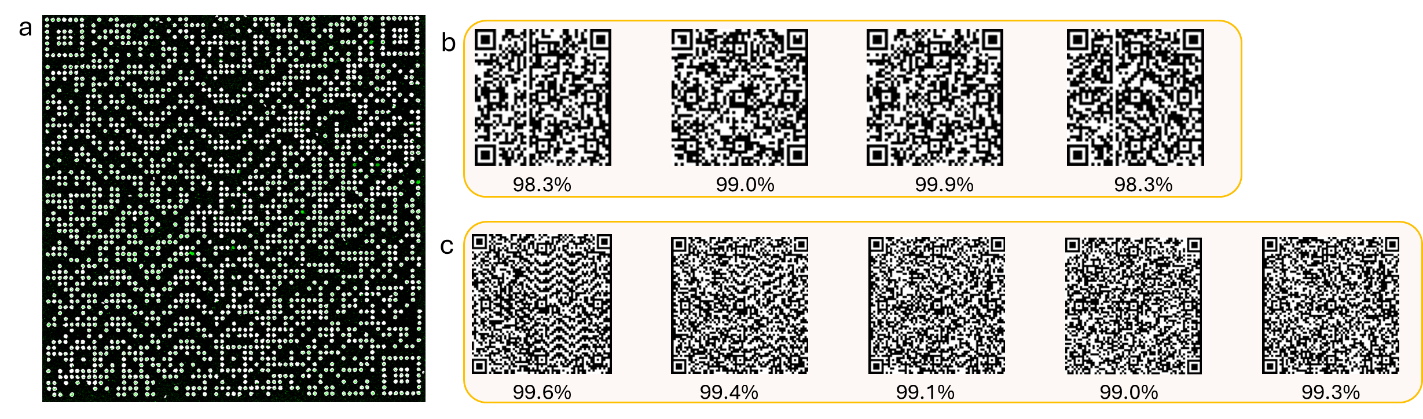


**Supplementary Figure S10. Recognition of QR codes.**

a. A representative example of direct recognition result through Python. The white and green dots represent successful and unsuccessful recognitons, respectively. b. The QR codes are converted by the fluorescent photos in Fig.3, along with a value of accuracy rate. c. The QR codes are converted by the fluorescent photos in Fig.5, along with a value of accuracy rate.

**Supplementary Table 1.** Different primer disks are used in this study. 5’-NH_2_ modified solid-phase primers were designed to modify the slide, forming a primer disk.

| **Primer disk** | **Primer Name** | **Primer Sequences** | **Figures** |
| --- | --- | --- | --- |
| 1-primer disk | PrimerT_NH_F1 | TTTTTTTTTTTTTTTACATTCCGTGCCATTGGATT | Figure 2c  Figure 3a  Figure 3b  Figure 5a  Figure S6 |
| 4-primer disk | PrimerT_NH_F1 | TTTTTTTTTTTTTTTACATTCCGTGCCATTGGATT | Figure 3c  Figure 4b  Figure 4c  Figure S5b |
|  | PrimerT_NH_F2 | TTTTTTTTTTTTTTTAATCATGGCCTTCAAACCGT |  |
|  | PrimerT_NH_F3 | TTTTTTTTTTTTTTTAATGGACGTTCCGCAATCAT |  |
|  | PrimerT_NH_F4 | TTTTTTTTTTTTTTTAAATCCTTTGTGCCTGCCAT |  |
| 5-primer disk | PrimerT_NH_F1 | TTTTTTTTTTTTTTTACATTCCGTGCCATTGGATT | Figure 5c  Figure 5d  Figure S7  Figure S8 |
|  | PrimerT_NH_F2 | TTTTTTTTTTTTTTTAATCATGGCCTTCAAACCGT |  |
|  | PrimerT_NH_F3 | TTTTTTTTTTTTTTTAATGGACGTTCCGCAATCAT |  |
|  | PrimerT_NH_F4 | TTTTTTTTTTTTTTTAAATCCTTTGTGCCTGCCAT |  |
|  | PrimerT_NH_F5 | TTTTTTTTTTTTTTTTGGCTCATTTCACAATCGGT |  |
| 10-primer disk | PrimerT_NH_F1 | TTTTTTTTTTTTTTTACATTCCGTGCCATTGGATT | Figure 2e |
|  | PrimerT_NH_F2 | TTTTTTTTTTTTTTTAATCATGGCCTTCAAACCGT |  |
|  | PrimerT_NH_F3 | TTTTTTTTTTTTTTTAATGGACGTTCCGCAATCAT |  |
|  | PrimerT_NH_F4 | TTTTTTTTTTTTTTTAAATCCTTTGTGCCTGCCAT |  |
|  | PrimerT_NH_F5 | TTTTTTTTTTTTTTTTGGCTCATTTCACAATCGGT |  |
|  | PrimerT_NH_F6 | TTTTTTTTTTTTTTTTTCGTTCGTCGTTGATTGGT |  |
|  | PrimerT_NH_F7 | TTTTTTTTTTTTTTTTCCTCAGCCGATGAAATTCC |  |
|  | PrimerT_NH_F8 | TTTTTTTTTTTTTTTTTTCGGAAGCCGCTTTGTAT |  |
|  | PrimerT_NH_F9 | TTTTTTTTTTTTTTTTTAATCGGTAACACCTGCGG |  |
|  | PrimerT_NH_F10 | TTTTTTTTTTTTTTTTCCATTGCGTCAACCGTTAT |  |

**Supplementary Table 2.** The primers of different DNA files used in this paper. The original files and encoded full sequences can be found in the supporting information.

| File No. | File Title | File Size | Forward Primer | Reverse Primer |
| --- | --- | --- | --- | --- |
| 1 | Badge.gif | 41 KB | TTTGTGGAACGATTTGCCGA | AATCCAATGGCACGGAATGT |
| 2 | GeneralInformation.pdf | 58 KB | AACAAGACTTTCGGAGCGTT | ACGGTTTGAAGGCCATGATT |
| 3 | Motto.jpg | 32 KB | ATTTACATTGCCACGGCTCT | ATGATTGCGGAACGTCCATT |
| 4 | States.txt | 18 KB | AATTGGTCGGCCTTGAGTTT | ATGGCAGGCACAAAGGATTT |
| 5 | TsingHuaCollegeSong.mp3 | 36 KB | TTGCACGGCAGGTCATTTAT | ACCGATTGTGAAATGAGCCA |

**Supplementary Table 3.** Comparison of our primer-disk approach and existing micro-disk approach.

|  | | **Primer Disk** | **Micro-Disk** |  |
| --- | --- | --- | --- | --- |
| Fabrication Steps | | 1. Preparation of primer disk: solve DNA into Na2CO3/ NaHCO3 solution, and cover the Isothiocyanate slide with solution 2. DNA files recording: binding in Flat PCR apparatus 3. Writing of fluorescent dot code: print the dot code and deposit at 20℃. Ink component: fluorescent DNA, ddH2O, Blunt/TA Ligase Master Mix (NEB) | | 1. DNA amplification and purification: link the primer for situ DNA production, and then purify DNA 2. DNA binding with prepolymer: DNA was mixed with the prepolymer resin at a 1:9 ratio by vigorously vortexing for 5 min at 2500 rpm. The prepolymer resin was prepared as a mixture of poly(ethylene glycol) diacrylate (PEGDA), poly(ethylene glycol) (PEG), and 3-(trimethoxysilyl)propyl acrylate (TMASPA, Sigma-Aldrich) as a alkoxysilane-grafted photocurable resin with Irgacure 1173 (BASF) as a photoinitiator. 3. DNA micro-disk synthesization: projecting the QR-patterned UV light on the slide. |
| Functions | | Read-many  Index  Record-many | Read-many  Index  Write-only |  |
| Density | | 10^12^ bit/mm^2^ | 10^12^ bit/mm^3^ |  |
