# Supplementary material for: Primer‐Disk‐Enabled DNA Data Storage System with Index and Record‐Many‐Read‐Many Features
Source: Adv Sci (Weinh). 2025 Jun 4;12(32):e02367. doi: 10.1002/advs.202502367 (PMC12407294; doi:10.1002/advs.202502367)
Supplement: Supplementary file 2 — Supporting Information [file ADVS-12-e02367-s002.zip › Original files and DNA sequences/GeneralInformation.pdf]

General Information

The campus of Tsinghua University is situated in northwest Beijing on the site of the former imperial gardens of the Qing Dynasty, and surrounded by a number of historical sites.

Tsinghua University was established in 1911, originally under the name “Tsing Hua Imperial College”. The school was renamed "Tsing Hua College" in 1912. The university section was founded in 1925. The name “National Tsing Hua University” was adopted in 1928.

The faculty greatly valued the interaction between Chinese and Western cultures, the sciences and humanities, the ancient and modern. Tsinghua scholars Wang Guowei, Liang Qichao, Chen Yinque and Zhao Yuanren, renowned as the "Four Tutors" in the Institute of Chinese Classics, advocated this belief and had a profound impact on Tsinghua's later development.

Following the outbreak of the War of Resistance against Japanese Aggression in 1937, National Tsing Hua University, National Peking University and Nankai University merged to form Lin-shih-ta-hsueh, which was renamed the National South-West Associated University in 1938 after moving to Kunming. In 1946 The University was moved back to its original location in Beijing after the war.

After the founding of the People's Republic of China, the University was molded into a polytechnic institute focusing on engineering in the nationwide restructuring of universities and colleges undertaken in 1952. In November 1952, Mr. Jiang Nanxiang became the President of the University. He made significant contributions in leading Tsinghua to become the national center for training engineers and scientists with both professional proficiency and personal integrity.

Since China opened up to the world in 1978, Tsinghua University has developed at a breathtaking pace into a comprehensive research university. At present, the university has 21 schools and 59 departments with faculties in science, engineering, humanities, law, medicine, history, philosophy, economics, management, education and art.

With the motto of “Self-discipline and Social Commitment” and the spirit of “Actions Speak Louder Than Words”, Tsinghua University is dedicated to the well-being of Chinese society and to world development. As one of China’s most prestigious and influential universities, Tsinghua is committed to cultivating global citizens who will thrive in today’s world and become tomorrow’s leaders.

Through the pursuit of education and research at the highest level of excellence, Tsinghua is developing innovative solutions that will help solve pressing problems in China and the world.
