# Supplementary figures and images for: Primer‐Disk‐Enabled DNA Data Storage System with Index and Record‐Many‐Read‐Many Features
Source: Adv Sci (Weinh). 2025 Jun 4;12(32):e02367. doi: 10.1002/advs.202502367 (PMC12407294; doi:10.1002/advs.202502367)

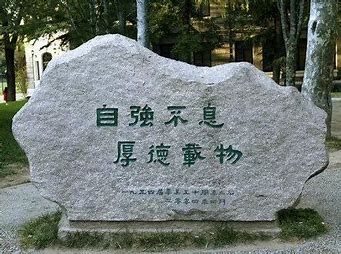

Supplement: Supplementary file 2 — Supporting Information [file ADVS-12-e02367-s002.zip › Original files and DNA sequences/Motto.jpg]

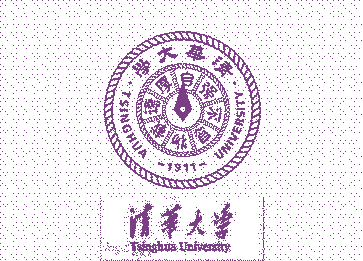

Supplement: Supplementary file 2 — Supporting Information [file ADVS-12-e02367-s002.zip › Original files and DNA sequences/Badge.gif]
